# Electrical and Photoelectrical Properties of Reduced Graphene Oxide—Porous Silicon Nanostructures

**DOI:** 10.1186/s11671-017-2043-7

**Published:** 2017-04-13

**Authors:** Igor B. Olenych, Olena I. Aksimentyeva, Liubomyr S. Monastyrskii, Yulia Yu. Horbenko, Maryan V. Partyka

**Affiliations:** 1 Department of Electronics and Computer Technologies (Сhair of Radioelectronics and Computer Systems), Ivan Franko National University of Lviv, 50 Dragomanov Street, 79005 Lviv, Ukraine; 2 Physical and Colloidal Chemistry Department, Ivan Franko National University of Lviv, 6 Kyrylo and Mefodiy Street, 79005 Lviv, Ukraine; 3 Solid State Physics Department, Ivan Franko National University of Lviv, 50 Dragomanov Street, 79005 Lviv, Ukraine

**Keywords:** Porous silicon, Reduced graphene oxide, Hybrid structure, Photosensitivity, Current–voltage characteristics, Impedance spectroscopy, 73.63.-b, 78.67.-n, 81.05.Rm

## Abstract

In this work, the hybrid structures were created by electrochemical etching of silicon wafer and deposition of reduced graphene oxide (RGO) on the porous silicon (PS) layer. With the help of SEM and AFM, the formation of hybrid PS–RGO structure was confirmed. By means of current–voltage characteristic analysis and impedance spectroscopy, we studied electrical characteristics of PS–RGO structures. The formation of photosensitive electrical barriers in hybrid structures was revealed. Temporal parameters and spectral characteristics of photoresponse in the 400–1100-nm wavelength range were investigated. The widening of spectral range of photosensitivity of the hybrid structures in short-wavelength range in comparison with single-crystal silicon was revealed. The obtained results broaden the prospects of application of the PS–RGO structures in photoelectronics.

## Background

The rapid development of electronics is associated with the search for new functional nanomaterials. Unusual properties of hybrid material combining graphene and porous semiconductors, in particular porous silicon (PS), have caused the emergence of potential applications, ranging from photoreceivers and biochemical sensors to field effect transistors and electrodes for power sources [1–5].

Graphene and graphene-based materials have been attracting great research interest with regard to their outstanding electronic and optical properties. Graphene is a two-dimensional sheet composed of sp^2^-bonded single-layer carbon atoms with the honeycomb lattice structure [6–8]. This conical shape of the electron spectrum of two-dimensional hexagonal lattice causes the most unique properties of graphene as a gapless semiconductor [9, 10]. In particular, the electrons in graphene behave like massless Dirac particles, and their mobility is almost two orders of magnitude higher than the mobility of free electrons in silicon [10–12]. In addition, the positions of the Fermi level and hence the concentration of free charge carriers in graphene are conveniently managed by external electrical voltage [10, 13]. With this approach, it is easy to inject carriers with positive or negative charge in graphene. Besides superior electric conductivity, graphene is characterized by high thermal conductivity and optical transparency [7, 12, 14]. One of the promising methods for producing graphene is via chemical exfoliation and oxidations of graphite to produce graphene oxide (GO) followed by subsequent reduction [15–17]. Reduced graphene oxide (RGO) is usually obtained through chemical reactions with the use of hydrazine, hydrogen, sodium tetrahydroborate, or other reducing agents [17, 18].

On the other hand, PS is recognized as an attractive material for photoelectrical devices because of high surface to volume ratio and high light absorption [19–22]. PS is prepared by etching a single crystal with the formation of small cavities and silicon nanostructures (nanowires and nanowalls) [23, 24]. Changing the band energy structure of silicon when moving from bulk to nanocrystals due to quantum confinement allows to significantly widen the absorption spectra of photonic devices based on the PS. Using multilayered photosensitive structures based on silicon nanomaterials allows to achieve a notable improvement in sunlight to energy conversion efficiency and to design the new generation photodetectors [25–27]. Also, PS is a perfect candidate for deposition and infiltration of graphene nanosheets and GO into sponge-like geometrical structure of the substrate [28, 29]. The photoreceivers with PS surface coated by RGO have high sensitivity and quantum efficiency over a wide spectral range—from near UV to IR [1]. Some previous researches have shown the effectiveness of using graphene as a transparent electrode in displays and photonic devices [14, 30, 31]. In this regard, the formation of PS–RGO structures is promising for their applications in photodetectors and energy conversion devices.

In this work, we created the hybrid PS–RGO structures in order to investigate their electrical and photoelectrical properties. Electrical parameters of obtained structures have been studied in alternating and direct current modes. It has been demonstrated that hybrid PS–RGO nanosystems have the potential for application in electronic and photoelectric devices.

## Methods

PS was manufactured by means of electrochemical etching performed in ethanol solution of hydrofluoric acid (the volume ratio of the components HF:C_2_H_5_OH = 1:1) on single-crystalline silicon substrates with the typical thicknesses of 400 μm and the crystallographic orientation (100). The silicon substrates had electronic type of conductivity (*n*-Si), with the specific resistance of 4.5 Ω cm. In order to obtain homogeneous layers of the PS, gold films were preliminarily deposited on a back surface of the substrates with the aid of a thermo-vacuum technique. These films served also as contacts for further measurements. The anodic current density was equal to 20 mA/cm^2^, and the etching time was 5–10 min. To ensure availability of holes in the surface layer of *n*-Si, which were necessary for occurrence of anodic reactions and formation of the PS, the working surface of a silicon plate was irradiated by 500-W filament lamp during the whole process of electrochemical etching [23]. As a result, narrow pores were formed that tend to direct towards the inside of the silicon crystal. After electrochemical processing, the working surface of sample was washed with distilled water and dried in air.

To obtain the hybrid PS–RGO nanostructures, graphene oxide produced by Biotool (Germany) in the form of an aqueous suspension (concentration of basic substance was 2 mg/ml) was used. A stable homogeneous suspension of the RGO was prepared by the reduction of aqueous dispersion of GO under action of hydrazine and treatment in an ultrasonic bath. The obtained RGO suspension was deposited onto a surface of porous layer and then dried at the room temperature during 48 h under dynamic vacuum.

Topology of hybrid PS–RGO structure was characterized by atomic-force microscope (AFM) “Solver Pro” and scanning electron microscope (SEM) “Selmi.” The electrical and photoelectrical properties of the samples were measured experimentally with standard techniques. The electrical current flowed through the structures in the direction perpendicular to their surfaces. The electrical parameters of the obtained nanosystems were investigated in both DC and AC regimes. The current–voltage characteristics (I-V curves) of sandwich structures based on PS were measured using AFM tip which was positioned on PS surface or RGO plate and using the indium tin oxide (ITO) contact, too. Impedance spectroscopy of the experimental samples was performed using R, L, and C measuring device E7-20 (“Kalibr,” Belarus) at room temperature in the frequency range of 25 Hz–1 MHz.

Photoelectric phenomena were investigated by irradiating the structures from the side of the porous layer with 1-W white LED (FYLP–1W–UWB–A) that provide light flux of 76 lm. Spectral dependencies of the photoresponse were measured using standard optical equipment, including a diffraction monochromator and a filament lamp (2800 K). Obtained spectra were normalized with respect to 2800 K black body radiation curve and were corrected taking into account the spectral sensitivity of our instrument. The spectral dependence of the photoinduced signal of an industrial silicon photodiode was also measured for a comparison. Kinetics of the photoresponse of hybrid PS–RGO structures to rectangular light pulses was examined using Hantek 1008B oscilloscope. UV–vis absorbance and transmittance spectra of the GO and RGO films on the glass substrate were measured in 220–1000-nm range using CM2203 spectrofluorometer (“Solar,” Belarus).

## Results and Discussion

**Fig. 1:**
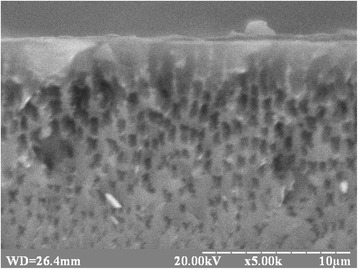
SEM image of the cross sections of the PS–RGO structures

Figure 1 shows the SEM image of cross section of the PS–RGO structures. Based on analysis of the SEM image, the sponge-like structure of PS layers was found. Average pore diameter was within the range of 100–1000 nm. The formation of macropores promotes to infiltrate of RGO nanosheets into porous layer.

**Fig. 2:**
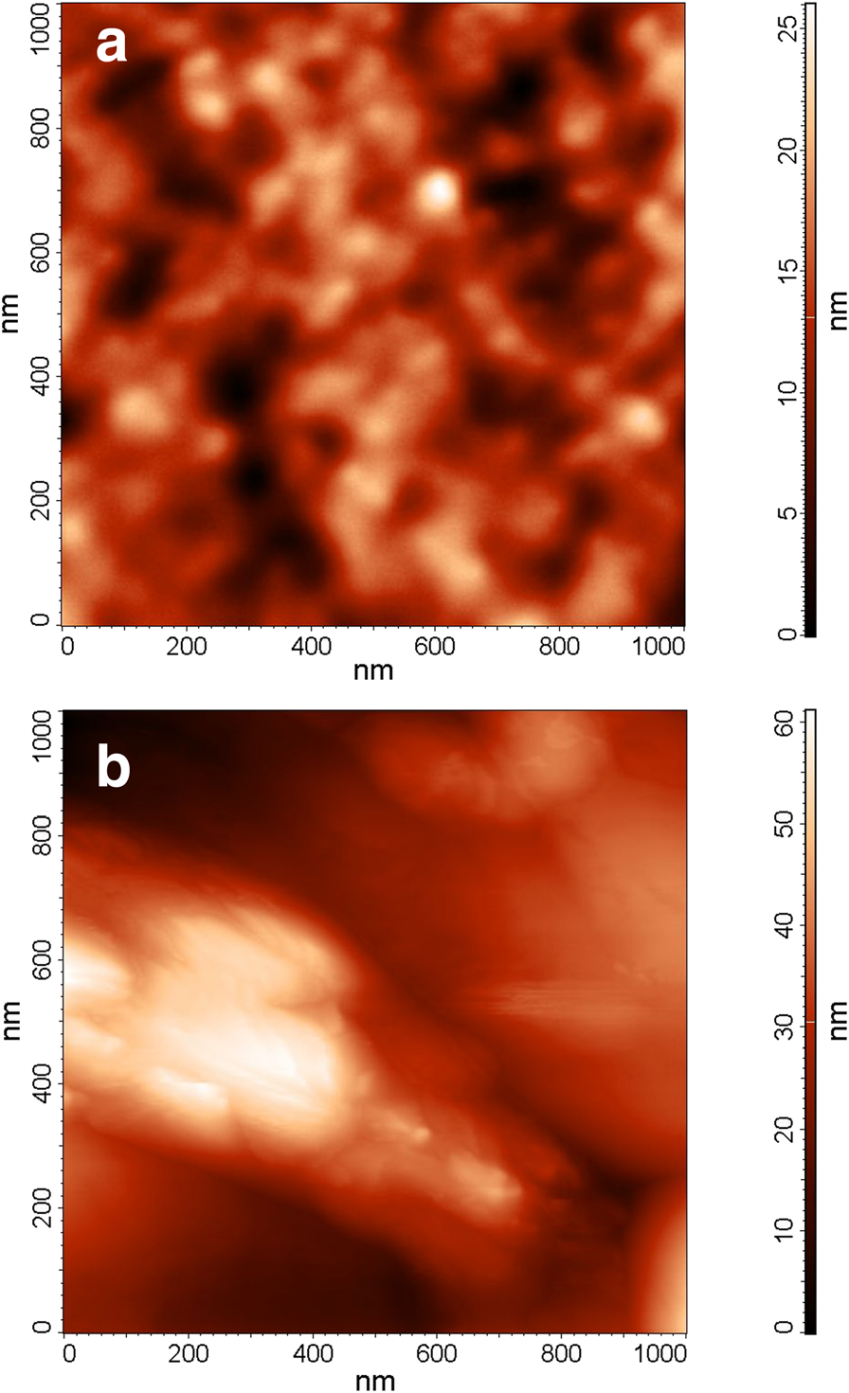
Two-dimensional AFM micrographs of the PS (**a**) and hybrid PS–RGO structures (**b**)

The surface topography of the PS-based structures was studied using AFM in the tapping mode. Figure 2 shows the two-dimensional micrograph of 1 × 1 μm^2^ area of the experimental samples. According to AFM data, PS nanocrystals had cross sections in range tens to hundreds of nanometers (see Fig. 2a). After the deposition of RGO, we observed the objects with the dimensions about several hundred nanometers on the PS surface (see Fig. 2b). We assume that these are RGO nanosheets.

**Fig. 3:**
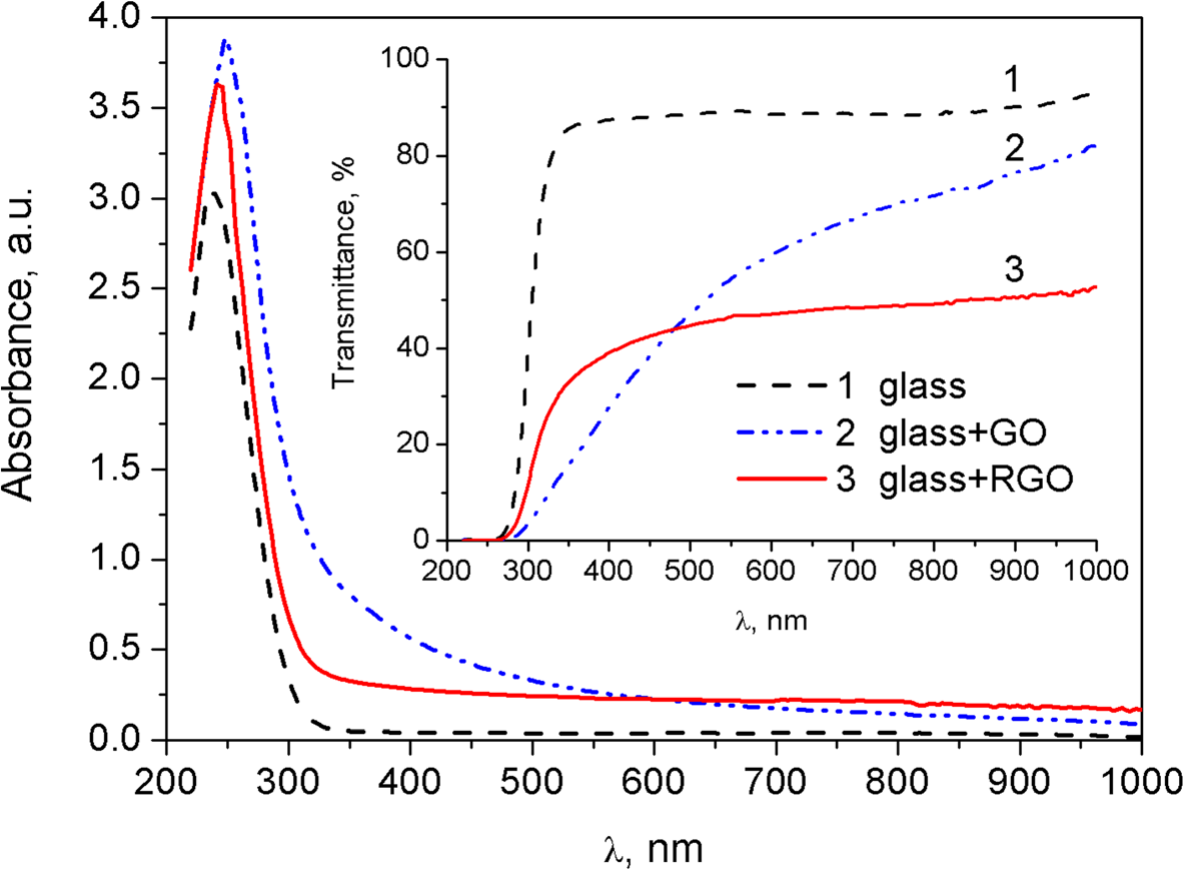
UV–vis absorbance and transmittance spectra of the glass substrate (curve *1*), films of GO (curve *2*), and RGO (curve *3*) on the glass substrate

Thickness of nanosheets in tens of nanometers, observed from Fig. 2b, can be caused by a multilayer structure of deposited RGO. UV–vis absorbance and transmittance spectra of RGO on the glass substrate that is shown in Fig. 3 indicate the partial aggregation of nanosheets, too. The decreased transmittance of the RGO films as compared to the glass substrate may be caused by the additional absorption and scattering of light by RGO multilayer. The peak in the 240–250-nm range can be related with the absorption in glass substrate. Therefore, further comparative analysis of UV–vis spectra of RGO and GO is based on the spectral information in the range of 300–450 nm only.

Meanwhile, we observed the decrease in light absorption in the RGO film as compared to GO in the near UV and blue part of the studied spectral range. It is usually attributed to *n* → π* transition of the carbonyl groups of GO [29, 32, 33]. This may indicate a high reduction degree of the GO in the RGO films. Such films have quite high optical transmittance in the visible region of the spectrum that allows their use as a protective coating and optically transparent electrode.

**Fig. 4:**
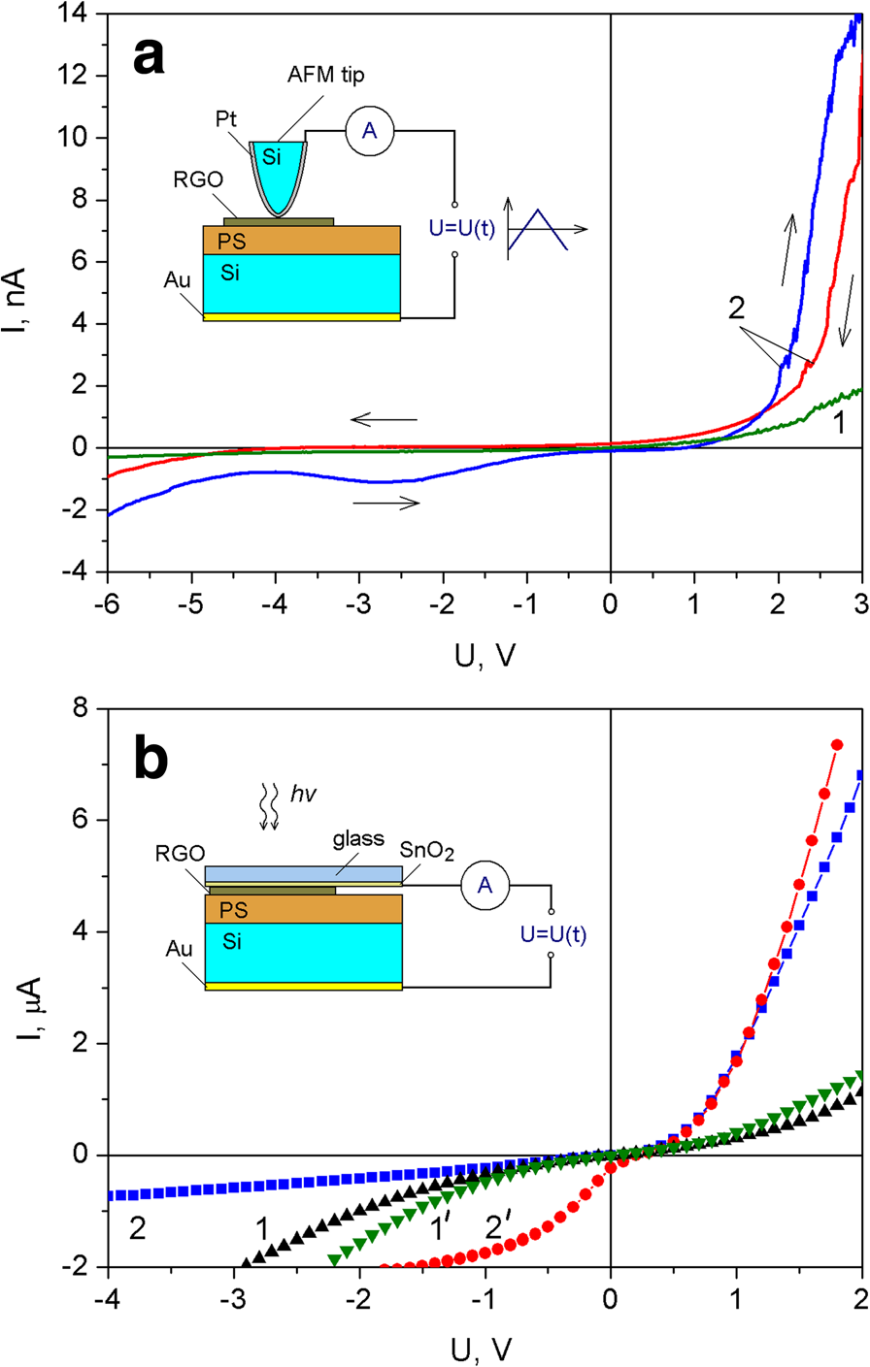
Dark I-V curves (curves *1* and *2*) and I-V curves after irradiation (curves *1’* and *2’*) of the initial PS (curves *1* and *1’*) and PS–RGO structures (curves *2* and *2’*) performed using the AFM tip (**a**) and the ITO contact (**b**). *Insets*: schemes of the I-V curve measurement for experimental structures

I-V curves of our structures based on the PS, measured at room temperature using AFM probe, are shown in Fig. 4a. The experimental samples were characterized with nonlinear I-V curves that can be caused by contact phenomena and electric barriers in the porous layer and on the interfaces of PS–silicon substrate and PS–PGO. Deposition of the RGO on the PS surface led to a change in electrical parameters of sandwich structures. We observed increase in conductivity and the rectifier-type I-V curves of the PS–RGO structures. Noticeable also is a diode-like nature of the I-V curves of the hybrid PS–RGO structures with ITO contact on surface of porous layer as well (Fig. 4b). Detected increase in electric conductivity of experimental structures with ITO contact can be connected with larger area of contact compared to AFM tip.

Under the influence of radiation with white light (76 lm flux), a photovoltage generation was observed and I-V curves of PS–RGO structures were changed similar to those of photodiodes (see Fig. 4b). The increase of the reverse current confirmed the photogeneration of free carriers in the structures under study. In the open-circuit regime, the photogenerated electron–hole pairs were separated at the potential barrier creating photovoltaic signal. The PS structure is characterized by lower photosensitivity compared to hybrid PS–RGO structures.

One has to note that infiltration of RGO nanosheets into PS can cause the formation of additional paths for current flowing through the porous layer and can ensure efficiency in collecting photogenerated carriers and extracting them from the bulk of PS layer.

**Fig. 5:**
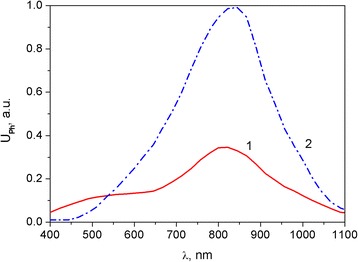
Photovoltage spectra for the RGO–PS structure (curve *1*) and industrial silicon photodiode (curve *2*)

Spectral dependencies of the photoresponse of hybrid PS–RGO structures are presented in Fig. 5. Photovoltage spectra in open-circuit regime were similar to the spectrum of the photoresponse of silicon diode and PS-silicon heterojunctions [34]. They are characterized by a wide maximum in the region of 750–950 nm. Apart from that, broadening of the photosensitivity spectral region towards higher energies for the PS–RGO structures was observed. It allows suggesting that the photovoltage is associated with light absorption not only in the silicon substrate but also in the PS nanocrystals.

**Fig. 6:**
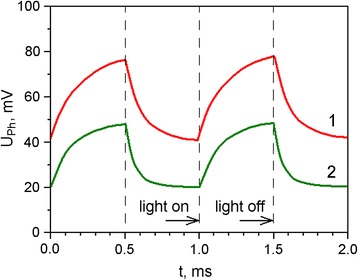
Kinetics of the photoresponse to rectangular 940 nm (curve *1*) and 570 nm (curve *2*) light pulses for PS–RGO structure

In order to get additional information about photoelectric processes in PS–RGO structures, their kinetic characteristics were investigated. Pulsed radiation of the IR and green light LEDs with wavelengths of *λ* = 940 nm and *λ* = 570 nm, respectively, were exploited. These wavelengths correspond to the photosensitivity bands of hybrid PS–RGO structures. Results of the study of temporal parameters of the photoresponse to rectangular 0.5 ms 1 kHz light pulses are shown in Fig. 6.

Analyzing the obtained dependencies, one can conclude that hybrid PS–RGO structures have slightly smaller photoresponse times to green light as compared to IR radiation. The observed temporal parameters of the photoresponse to light pulses of different wavelengths can serve as an additional argument in favor of our hypothesis that various layers of the hybrid structure are responsible for the absorption of light quanta of different energy.

**Fig. 7:**
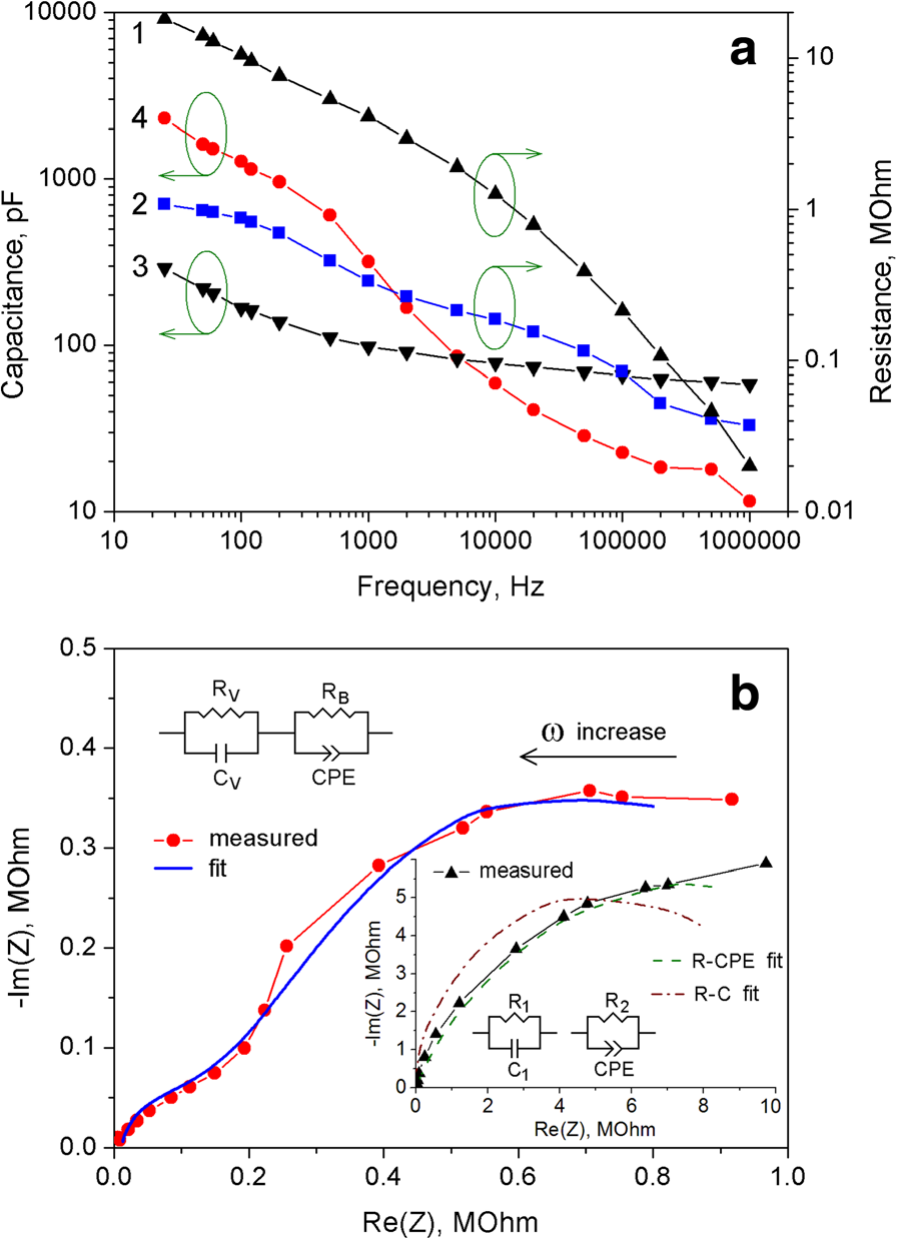
**a** Frequency dependence of the electrical resistance (curves *1* and *2*) and the capacitance (curves *3* and *4*) of the PS (curves *1* and *3*) and PS–RGO structures (curves *2* and *4*). **b** Nyquist plot and equivalent circuit diagram of PS–RGO structure. *Inset*: Nyquist plot and equivalent circuit diagrams of the PS structure

In order to study the influence of RGO deposition on the electrical properties of the PS, we have investigated the frequency dependences of impedance of obtained structures. Impedance spectroscopy is a powerful technique for the study of the processes of charge transfer in the nonhomogeneous and fractal systems [35–37]. By means of impedance spectroscopy, it was found that our structures based on the PS show a decrease in electrical capacitance and internal resistance with increasing the frequency (Fig. 7a). In addition, the PS–RGO structures have less resistance and greater capacity compared to PS structures in the low-frequency range. This may be caused by infiltration of the RGO into porous layer.

Figure 7b presents Nyquist plots of the structures based on PS in the complex plane *Z*_Re_–*Z*_Im_. To interpret the impedance of experimental structures, the equivalent circuit models were constructed. Within the first model for the PS, the porous layer can be regarded as the composition of parallel-connected capacitor with the capacitance *C*_1_ and resistor with the resistance *R*_1_ [29]:

$$ Z\left(\omega \right)=\frac{R_1}{1+ j\omega {R}_1{C}_1}. $$

After the approximation of the impedance spectra of the PS structure, the following values were derived: *R*_1_ = 10.3 MΩ and *C*_1_ = 177 pF. In the second model for the PS, the resistive–capacitive properties of the system are described using the constant phase element (CPE) in the equivalent circuit as shown in inset Fig. 7b. Expression for the impedance of the R–CPE model will be the following [38]:

$$ Z\left(\omega \right)=\frac{R_2}{1+{\left( j\omega \right)}^n{R}_2 Q}, $$

where *Q* is the CPE and *n* characterizes the heterogeneity of the electrical properties of the structure (−1 ≤ *n* ≤ 1). In case *n* = 1, CPE corresponds to the pure capacitance, and when *n* = 0, CPE transforms into the simple active resistance. In case of the R–CPE model, the approximation parameters of the PS impedance are *R*_2_ = 16.4 MΩ, *Q* = 9.8 × 10^−10^ and *n* = 0.74. Analysis of the results shows that the electrical properties of the PS nanostructures are described better using the CPE in the equivalent circuit.

According to the model for the PS–RGO structure, the total impedance *Z*(ω) of the sample can be represented by two *RC* (parallel resistor and capacitor) sub-circuits connected in series. Moreover, low-frequency *R*_*B*_ *C*_*B*_ and high-frequency *R*_*V*_ *C*_*V*_ sub-circuits correspond to the process of transfer of charge carriers through the boundary and in the bulk of silicon nanocrystals, respectively. For the analysis of this model, it is also appropriate to use the CPE. After replacing the *C*_B_ capacitor by CPE, the expression for the impedance of the PS–RGO structures will be the following:

$$ Z\left(\omega \right)={R}_0+\frac{R_V}{1+ j\omega {R}_V{C}_V}+\frac{R_B}{1+{\left( j\omega \right)}^n{R}_B Q}. $$

As it is evident from Fig. 7b, the contribution of active resistance *R*_0_ of the silicon substrate and the supply contacts is negligibly small (real axis value intercept at *ω* = ∞ is about zero). Therefore, we do not consider this resistance in the impedance models for experimental PS–RGO structures.

Based on the approximation of the impedance spectra of PS–RGO structures, *R*_*V*_ and *R*_*B*_ values were determined to be 0.41 and 1.01 MΩ, respectively. Capacitance *C*_*V*_ was about 5.1 nF. Parameters of CPE were *Q* = 1.27 × 10^−7^ and *n* = 0.37. We envisage that graphene sheets provide better connection between PS nanocrystals to improve electrical conductivity of PS–RGO nanosystem. This expands the prospect of hybrid structures as active materials for storage and conversion of energy.

## Conclusions

In this study, we focused on novel technical solutions relevant for the design of photosensitive structures based on porous silicon. Photosensitive hybrid PS–RGO structures were created by the method of electrochemical etching of silicon wafer and deposition on the porous silicon layer of reduced graphene oxide prepared from water dispersion. Sponge-like morphology of PS favors the incorporation of RGO nanosheets into its bulk forming additional pathways for current flow through the porous layer. As a result, there are changes in electrical parameters of PS structures.

The internal resistance and electrical capacitance of PS-based structures were determined using the approximation of the impedance spectra. It was shown that the impact of RGO on electrical characteristics of PS manifests a reduction (about and order in magnitude) of the internal resistance of hybrid structures. By means of impedance spectroscopy, it was found that experimental structures show a decrease in electrical capacitance and internal resistance with increasing the frequency from the 25 Hz to 1 MHz.

The photovoltaic processes in hybrid nanostructures have been studied by means of comprehensive research of I-V curves and spectral and temporal dependencies of photoresponse of PS–RGO structures. The hybrid PS–RGO structures demonstrate the photovoltaic effect in the wide spectral range but have some peculiarities. Beside the intense band near 820 nm, the weak band of photosensitivity is observed in 500–600-nm range. It was found that the rise time and attenuation of photoinduced signal of experimental structures depends on the wavelength of the light pulses and is about 1 ms. A large surface area of PS provides effective absorption of light quanta of different energy and hence high photosensitivity of hybrid structures over a wide spectral range. The obtained experimental results can be used for the design of photodetectors and other optoelectronic devices based on PS nanocrystals.

## Abbreviations

AFM: Atomic-force microscope
CPE: Constant phase element
GO: Graphene oxide
IR: Infrared
ITO: Indium tin oxide
I-V curve: Current–voltage characteristic
LED: Light-emitting diode
PS: Porous silicon
RGO: Reduced graphene oxide
SEM: Scanning electron microscope
UV: Ultraviolet
